# “They should’ve talked to us more”: lay health advisors’ experiences with community-engaged hypertension research

**DOI:** 10.1186/s40900-024-00544-8

**Published:** 2024-01-25

**Authors:** Cyleste C. Collins, Mona Shediac-Rizkallah, Jacqueline Dolata, Erika Hood, Elodie Nonguierma, Daryl Thornton

**Affiliations:** 1 School of Social Work, Cleveland State University, 2121 Euclid Ave, RT 1438, Cleveland, 44115 OH USA; 2 Center For Health Equity, Engagement, Education, and Research, MetroHealth Medical Center, 2500 MetroHealth Drive, Cleveland, OH 44109 USA; 3 Population Health Institute, MetroHealth Medical Center, 2500 MetroHealth Drive, Cleveland, OH 44109 USA; 4 See You at The Top, Cleveland, OH USA

**Keywords:** Lay health advisors, Hypertension, Patient and public involvement, Community-engaged research, Qualitative study

## Abstract

**Background:**

Lay health advisors (LHAs) are increasingly being used to increase patient and public involvement in research, disseminate health information, and work toward preventing health disparities within communities at risk. This research explored LHAs’ experiences with training and recruiting for a hypertension research project which ended due to minimal enrollment.

**Methods:**

The methodological design was qualitative description. One face-to-face semi-structured focus group was held with eight African American LHAs in Cleveland, Ohio, in the fall of 2019. The focus group was digitally recorded and transcribed by a professional transcriptionist and thematically analyzed.

**Results:**

Trainees reflected on how much they learned from the training and described feeling passionate and excited about their community work for the project. We identified three key themes from the data: (1) Systemic and Institutional Factors Affected LHAs’ Experiences (subthemes: Unnecessarily Burdensome Requirements and Exploitation of Community Members for Research Gain; (2) Feeling Used Yet Unseen: Exclusion from Decision-Making Processes; (3) Worrying that Project Termination Damaged their Reputation; and (4) Disengaging from Research. We share lessons learned, including the need for LHAs’ expertise to be integrated into research studies, and for projects to establish clear communication and expectations regarding research rigor and requirements.

**Conclusion:**

Our results have implications for future studies attempting to build equitable and strong academic-community relationships to yield rigorous and useful research to reduce health disparities.

## Abstract (plain language summary)

Community health workers (CHWs) are usually community members trained broadly to help the community address health issues. Community members (including patients and the public) who have experiences with specific diseases can be important resources in health research, contributing critical on-the-ground feedback and information about and to their communities, especially those with high rates of health disparities. In this study, CHWs with personal experience as patients with hypertension were trained to become lay health advisors (LHAs) specifically to assist with a hypertension research study in Cleveland, Ohio. This study explored LHAs’ experiences with their training for the research study and the study itself, which ended early because the project did not get enough participants. Eight African American LHAs participated in a focus group in the fall of 2019, sharing their experiences. The focus groups were recorded on a digital device and a professional transcriptionist transcribed them. We found that the LHA trainees learned a lot from the training. They were passionate and excited about working with the community work for the project, but also felt used by the project leadership, and not included in important decisions. When the project ended, they also felt their reputations were damaged. Finally, the experience with the project led to LHAs wanting to not participate in research anymore. We conclude that projects should include strong and clear lines of communication, better integrate LHAs into research studies as partners and honor their expertise.

## Background

In recent decades, comprehensive and participatory research approaches have been emphasized as ideal in addressing complex social determinants of health and health disparities in marginalized communities. When specific disparities affect particular populations, conducting culturally tailored, community-informed research in the affected communities is essential for developing a strong understanding of the phenomena [1]. Community-engaged research, including communities (patients and the public) in research, is increasingly understood as an effective way to understand specific disparities and work toward health equity. When communities are involved in research, researchers and communities engage in co-learning, however, community-engaged research is time and resource-intensive even for experienced community-engaged researchers [2]. This study explores community members’ experiences being trained as lay health advisors (LHAs) to assist with a randomized controlled trial (RCT) run by an established health disparities research center highly experienced with community-engaged research.

### Facilitators and barriers to successful community engagement in research

Tensions between academic researchers, lay community advisors, and/or program planners are common, as each group has different perspectives and priorities [3]. For example, community members might not understand or accept research institutions’ regulations and requirements (e.g., institutional review boards, long approval processes in research budget offices, etc.). Likewise, academics do not necessarily always know the community’s concerns, engage community members effectively in research, or understand the benefits of doing so [4]. Academics’ training, location in the university, and other factors often separate them from the community context, which can lead to biased and locally and culturally inappropriate research designs, conceptual models, and study instruments [5–7]. In some community-engaged research models, the community might provide some input on selecting the research question(s), but academic researchers determine methods of inquiry and the range of acceptable responses [8, 9]. In other models, the community contributes to other (or all) research phases [10].

Academics also often have little understanding of the most effective ways to engage in relationships with the community. The separation of communities (patients and the public) and academics can lead to superficial [11] “drive-by research” or “helicopter” relationships in which academic investigators come in, collect data, and disappear without giving back to the community [11–14] that leads to communities mistrusting academics and research. History is replete with examples in which researchers exploited Black participants (e.g., the Tuskegee Syphilis Study). Knowledge of, and even indirect exposure to, such events can lead to mental and physical health consequences, a phenomenon referred to as “peripheral trauma” [14] and a well-founded mistrust and skepticism of research and researchers [15].

### Lay health advisors

Integrating lay health advisors (LHAs) into research is one way to bridge gaps between researchers and community members. LHAs tend to have similar experiences and backgrounds as the people in the community with which they work, and while they are not professionals, they have undertaken specialized training beyond most lay people [16]. Because of their specialized training, the community may view them as outsiders [16]. However, systematic reviews have found them effective in providing support for under-served populations [17]. LHAs have been used across the United States on health issues as diverse as cancer prevention [18, 19], reducing cardiovascular risk [20–23], and sexually transmitted diseases [24]. Research evidence also suggests LHAs may help encourage preventive healthcare. For example, one RCT found that low-income African American patients receiving an LHA intervention were more likely to obtain mammography screenings than a comparison group [23].

A systematic review of LHA interventions identified best practices, including supporting LHAs’ leadership development and integrating them into the research design, research process, and meaning-making of research findings [20]. The review identified LHAs’ unique contributions as contextualizing findings and helping to determine the feasibility of community-based research efforts. Research on LHAs’ experiences suggests contextual and organizational factors, especially “outer contextual factors” are important for program impact and sustainability [25]. Outer contextual factors include policies, organizational partnerships, and external funding, which affect “inner contextual factors.” Inner contextual factors, meanwhile, include implementation processes, intervention characteristics, characteristics of those delivering the intervention, leadership/program champions and resources (e.g., funding), and implementation processes (e.g., communication, planning, training, and recruitment). According to the review, LHA characteristics included role commitment and clarity, role self-efficacy, and offering a stipend or payment. Important intervention characteristics included adaptability and perceived benefit and need.

In the current study, LHAs were specially trained community members focused on addressing social determinants of health related to hypertension within their communities. While we know much about the benefits of LHAs in distributing public health information and education in communities, we know little about LHAs’ qualitative experiences with RCTs.

### Description of the RCT

In 2017, the Center for Reducing Health Disparities in Cleveland, Ohio was awarded a National Institutes of Health (NIH) U54 grant Involving Communities in Delivering and Disseminating Health Disparity Interventions. One study from that grant was Using Lay Health Advisors to Help African Americans Address the Social Context of Hypertension Management, a 3-year cluster RCT for African American patients with poorly controlled hypertension [26]. Based on past Center for Reducing Health Disparities research that found better outcomes for patients engaging with health navigators [27], the study was designed to have intervention group patients meet with LHAs monthly and the control group receive treatment as usual. The researchers hypothesized that the intervention group would demonstrate lowered blood pressure, changes in hypertension knowledge and perceptions, increased medication adherence, more positive healthcare interactions, and improved quality of life. The LHAs were expected to help study patients better utilize their social networks to access health care, social services, and community resources, and teach patients to communicate with their primary care physicians more effectively about their barriers to hypertension management.

Although CHW and LHA are sometimes used interchangeably, in this study, LHAs were defined as persons with formal CHW certification who also had personal experience as hypertension patients, like the RCT’s target research participant pool. The LHAs were recruited from the same neighborhoods as the health system’s clinics, and were at least high school graduates, had hypertension themselves, were in good health and able to drive to meetings, worked flexible hours, had good communication skills, and had community volunteer or leadership experience.

By the time the project began, the intended primary investigator for the study had moved on, could no longer lead the study, and new project staff were assigned to it. LHAs were onboarded in the spring of 2018 and training began in the fall of 2018. LHAs attended six weekly training sessions held for 2 h in the evening at the Center for Reducing Health Disparities. The training session agendas are posted in Table 1. The training, designed to be culturally competent, integrated an African American heritage framework to educate patients about blood pressure and hypertension treatment. The project’s primary investigator and Center for Reducing Health Disparities staff, all of whom were African American, designed and facilitated the training. Training topics included basic overviews of the study and research, hypertension, hypertension’s social and cultural context, hypertension management, motivational interviewing, and more. Expert guest speakers were brought in to discuss hypertension management, cognitive behavioral therapy, smoking cessation, motivational interviewing, and interventions to improve food access in food deserts. By the fall of 2019, when we held the focus group, the LHAs were supposed to have been hosting sessions with intervention group patient participants, but the spring 2019 sample size was way below what the project team had projected, and the funder (NIH) asked the team to pivot because the project was not meeting its recruitment goals.

**Table 1:** Training session topics

Session	Topics covered	Learning objectives
1 Study and Hypertension Overview	Welcome	Describe research study purpose and details
	Center and Study Overview	Understand lay health advisor role and responsibilities
	Lay Health Advisor Introduction	Define hypertension
	Lay Health Advisor Role and Responsibilities	Describe hypertension symptoms, how it is diagnosed, and treatment options
	Research Overview	
	CREC, Human Subjects Protection, and Informed Consent	
	Hypertension Overview	
	Definition, Diagnosis, Pharmacologic and Non-Pharmacologic Treatment	
2 Socio-Cultural Factors and Barriers to Hypertension	Session 1 Review	Describe patient, provider, and system level barriers to hypertension management
	Cultural and Social Context of Hypertension	Describe socio-cultural barriers to hypertension management
	System, Provider, Patient, and Socio-cultural Barriers to Hypertension Management	Identify strategies to address barriers to hypertension management
	Hypertension Management Strategies	
	Blood Pressure Measurement training	
3 Behavioral Models and Patient Engagement	Session 2 Review	Describe behavioral models and techniques used to manage hypertension
	Motivational Interviewing	Describe 2 adult learning styles
	Cognitive Behavioral Change/Smoking Cessation	Demonstrate how to develop SMART goals
	Adult Learning Styles	
	Goal Setting (SMART Goals)	
4 Healthy Lifestyle and Community Resources	Session 3 Review	Describe the relationship between food environment and hypertension management
	Nutrition	Understand the benefits of healthy eating and active lifestyles
	Active Living/Exercise	Describe the services offered by United Way 211
	Stress Management	Identify community resources in the Greater Cleveland area
	Community Resources	
5 and 6 Group Facilitation and Conflict Management	Session 4 (and 5) Review	Describe the components of effective participant engagement
	Effective Communication	Understand the role of storytelling and narrative theory
	Key Techniques for Effective Facilitation	Describe problem solving and conflict management techniques
	Storytelling	
	Problem Solving	
	Conflict Management	

### Aims and research questions

Because LHAs are important to educating the community and helping people get the care they need, it is important to build knowledge about how they might contribute to research, best practices to prepare them to engage in research, and the factors that influence their experiences engaging with research. This study’s purpose was to explore the LHAs’ experiences with their training and experiences with the research project. The research questions were: (1) What were LHAs’ experiences with their LHA training? (2) What were LHAs’ experiences with the hypertension RCT? (3) What factors influenced LHAs’ experiences?

## Methods

### Design

We employed qualitative description (QD), a pragmatic approach, and used a social constructionist framework to learn about participants’ experiences [28–31]. This design was appropriate because we hoped to describe and explore participants’ experiences with the training and research and the meaning of those experiences. A focus group was the ideal data collection method. First, the LHAs knew each other well and shared the same experiences with the training and research project. Second, we were interested in understanding the LHAs’ understandings and experiences as a group.

### Participants

Eleven LHAs went through the full onboarding process, two dropped out before the study’s end (because of family constraints and a new job), and eight were present in the focus group. All were African American, were in their mid-forties, and all but one was a woman (see Table 2). Of the focus group participants, seven were women, and one was a man. More than one-third had either some college or an associate degree, a little more than a quarter had a bachelor’s degree, and more than one-third had some post-graduate education or a graduate degree. The LHAs had a variety of community involvement experiences before the study. These included working with their churches, as members of public health associations (state and national), school ambassadors or liaisons, citizen advisory committees, grassroots organizations addressing policy issues, community development partnerships, block parties and street clubs, literacy programs, elections, shelters, youth engagement, doula services, community organizing, youth ministry, assisting seniors in navigating services and policies, addressing barriers to care through food banks, prison re-entry programs, helping people with transportation, connecting people to services, providing health education, assisting with health screenings.

**Table 2:** LHA demographics

	*N* = 11 (%)
Gender	
Women	10 (90.9%)
Men	1 (9.1%)
Age (M (SD)), Median	44.4 (6.7), 46
Education	
Some college or associate degree	4 (36.4%)
Bachelor’s degree	3 (27.3%)
Some post graduate education or graduate degree	4 (36.4%)
Black or African American	11 (100%)

### Focus group interview guide

The first author and program staff collaborated in developing the focus group interview guide. The 11 focus group questions are included in Table 3. In the first part of the focus group, the questions asked participants to reflect upon the training and experiences with the project overall, including what they learned, what was most and least helpful in the training, how they would suggest improving it in the future, and what they would tell others who might be interested in the training about what to expect. They were also asked to compare their CHW training with the LHA training (e.g., what the LHA training added to what they already knew). In the second part of the focus group, participants were asked to think about their interactions with patients on the project, including how prepared they felt, what they were uniquely contributing, and what they liked best about working with the patients and what was challenging. Finally, time was allowed for the participants to offer any other comments.

**Table 3:** Focus group questions

Intro: “The questions that follow refer to your Lay Health Advisor (LHA) training and experiences so far in the project.”
Prompt: “Thinking back to your training….”
1. What would you say you learned from undergoing the training?
2. What part(s) of the training were most helpful for you?
3. What part(s) of the training were least helpful for you?
4. What would you change about the training and/or how would you recommend it be improved for the future?
5. What would you tell someone who was interested in participating in the training about what to expect?
6. What other comments and/or thoughts do you have about your training experience?
Prompt: “Thinking about your interactions with patients on the project…”
7. Given your background as a community health worker, how would you describe the LHA training as similar to or different from that training?
8. How did your previous experiences prepare you for interacting with patients? What do you feel like was your unique contribution?
9. What kinds of recruitment and engagement strategies worked best for you in connecting with patients?
10. What did you like best about working with the patients? What was challenging?
11. What else would you like to share about your experiences as an LHA?

### Procedures

#### Data collection

The MetroHealth Institutional Review Board (#IRB18-00038) approved all human subjects protocols and participants completed informed consent documents to participate in the study. The focus group was intended to be held mid-project to learn how the training had prepared LHAs to work on the research project, however, the research project could not be carried out as planned because the study’s recruitment strategies were not yielding enough participants. The focus group was held in the fall of 2019, on the same day the LHAs learned about the project ending. The focus group was conducted in a conference room at the training site. The focus group lasted 1 h and 15 min. The first author, a Ph.D. level evaluator with extensive experience in qualitative research and many years’ experience conducting focus groups with community members, facilitated the focus group and recorded it with a digital voice recorder. A professional transcriptionist transcribed the focus group verbatim.

### Establishing qualitative data trustworthiness

Data trustworthiness (credibility) included debriefing sessions, developing familiarity with the organizational culture, tactics to ensure participants’ honesty, and iteratively questioning the participants [32]. The first author had debriefing sessions with the project managers to check in on the training sessions’ progress and the project’s timeline. She also visited the LHA group once at the beginning of their training to collect information about their beliefs about the causes of hypertension. Thus, the first author gained familiarity with the trainees before the focus group data were collected, was not a total stranger to the group, but was not officially a project team member. This outside position might have enhanced the participants’ authenticity and willingness to be open and honest about their experiences [32]. She had been involved in research with the Center for Reducing Health Disparities for seven years before the focus group was conducted. During the focus group itself, iterative questioning helped clarify participants’ points and perspectives. Finally, source triangulation [29] was established by reviewing project documents, including the initial grant proposal, timelines, curriculum materials, calendars, and the LHA training manual. We conducted a member check with one LHA, who reviewed this manuscript and offered positive feedback.

### Analysis

Consistent with Braun and Clarke’s thematic analysis approach [33], the analysis began with the analyst focusing on immersing in the data by reading the transcripts at least twice. Then, inductive coding began, in which the analyst examined the transcripts line-by-line, highlighting transcript passages, including relevant context, and creating a code that seemed to capture the passage’s meaning. Once the entire transcript was reviewed and coded in this way, the codes were reviewed together, grouped and re-grouped, and themes were developed that encompassed multiple codes. The analyst reviewed the themes, defined and reviewed them, and identified quotations and passages that were good examples (exemplars) of the themes. The first author then distributed a detailed report including codes, themes, and exemplars, to the four-person project team to check against the analyst’s interpretations and any biases that might have arisen in the analysis process. After receiving no feedback regarding the interpretations in the report, the first author drafted a final report to the team. The team then met on Zoom to further discuss and finalize the themes they felt the quotes and codes reflected.

## Results

Below, we first discuss the LHAs’ experiences with the training, and then the main themes we identified representing their experiences with the research project: (1) Systemic and Institutional Factors Affected LHAs’ Experiences; (2) Feeling Used Yet Unseen: Exclusion from Decision-Making Processes; (3) Worrying that Project Termination Damaged Their Reputation; and (4) Disengaging from Research. The first theme included two sub-themes, Unnecessarily Burdensome Requirements, and Exploitation of Community Members for Research Gain. Table 4 includes the themes and representative quotes.

**Table 4:** Focus Group Themes, Sub-themes, and Representative Quotes

Theme	Sub-theme	Representative quote
Systemic and Institutional Factors Affected LHAs’ Experiences	Unnecessarily Burdensome Requirements	“You got to wait on the IRB. Time is not waiting on anybody, and time is constantly moving.” “All that stuff interfered with what we needed to get out here to be effective as we could be.”
	Exploitation of Community Members for Research Gain	“We are only there to still be that African American face.” “It’s money. You don’t care about what we are and what we do and who we are.”
Feeling Used Yet Unseen: Exclusion from Decision-Making Processes	N/A	“With us already being in the community, I think they should’ve talked to us more. …We know the people that’s out there.” “We didn’t even get a chance to get it off the ground or make mistakes.”
Worrying that Project Termination Damaged Their Reputation	N/A	“We’re representing the whole project, and we had to go on this road because we believed that it was gonna go somewhere and be for the greater good.” “Not only will we run into them, they’re gonna remember us.”
Disengaging from Research	N/A	“I’ve never been in a position like this, or doing research, or being part of a research team, and it’s kind of a disappointment.” “Scrap these institutions.” …We’re all capable of teaching people in our community.”

### The training: a learning experience

The LHAs explained why the training had appealed to many of them, saying they felt it furthered their skills and could open future job possibilities.

I think LHA is another level up from CHW, even though they say they’re all the same, but LHA is like, for example, the CDC, they hire LHAs. They don’t hire community health workers, so and they pay an LHA a lot more.… I’m actually operating under the title of LHA, so if I happen to go to the CDC and want to work with them, then I have the training to do it under that title.

The LHAs were positive about the training facilitators, saying they were dedicated and attentive. The LHAs felt the training increased their hypertension knowledge but also thought the training’s structure and curriculum should have been more formal. Participants said the training covered some information they already knew, and some they did not. For example, they said they were surprised to learn how many young people are affected by hypertension and how food intake, including salt and alcohol consumption, affect hypertension.

You find out how very little you may know… I only knew *of* hypertension, even though I had it. I didn’t know as much as I’ve learned about when I came here. So it was to my advantage, and to the advantage to use… in the community.

The LHAs also said they learned more about medications. “I didn’t know the different formulas of the medications, and how they have the different side effects, or how one may work with one medication, or if you have certain allergies, how this one wouldn’t work well for you.” They were eager to teach the community about various topics, including why people should not half their medication doses to stretch it out. The LHAs said their learning occurred during the training but also while assisting patient participants and doing “community pop-ups” (i.e., education sessions). They said they learned people should not talk when having their blood pressure taken and make sure their feet are on the floor. Another participant said he/she was surprised by how many patients did not have equipment for home blood pressure monitoring.

The LHAs said the training’s speakers–especially the physicians–helped increase their knowledge. They said discussions they learned about how doctors are utilizing food prescriptions and “how you could use your food stamps and double your purchase at certain locations.” The LHAs said academic and other lectures were useful:

Even the opportunity to go to some of the community conversations that were being held by different institutions.… Conversations (around) racism, or health, or any type of disparities were really good to see how it really affects your health and how it could affect the hypertension.

Overall, the LHAs said the training helped them to develop a deeper understanding of hypertension they said would be useful in their community work.

### Theme 1: systemic and institutional factors affected LHAs’ experiences

The LHAs discussed systemic and institutional barriers both to the project’s success and to their own experience, which they felt hampered their work on the project.

#### Subtheme 1: unnecessarily burdensome requirements

The LHAs were frustrated at having to comply with the large hospital system’s employment requirements. Just before the focus group began, the project director had reminded them they were required to get a flu shot, which the LHAs brought up as an example of a barrier, feeling it was unnecessarily burdensome since they did not work full time or with patients. One said: “You’re gonna make us get a flu shot? We don’t work in the hospital.”

Other institutional barriers the LHAs mentioned included dealing with the Institutional Review Board (IRB), completing effort reports, and spending their own gas money on project tasks. One participant summed up their frustrations with institutional barriers, saying they were directed to “Do this. Do that. Do this.” One LHA said: “We did the training, and at the same time, IRB and whatever technical stuff you have to get done, all that stuff interfered with what we needed to get out here to be effective as we could be.” They said the IRB application review process and technical training took too much time, keeping them from their work.

#### Subtheme 2: exploitation of community members for research gain

The LHAs were disappointed in part because they were excited about the work on an issue they recognized as a “silent killer” for which research was desperately needed. One said, “It’s money. You don’t care about what we are and what we do and who we are as people. We’re research projects. We are research for them to (take your) stem cells, womb.” Referencing Henrietta Lacks, the same LHA said, “It’s the 2019 version of that.” The LHAs’ comments reflected a broader disillusionment with research as a larger structural force perpetuating systemic racism and their concerns that they were being used. One quote from the group emphasized these ideas:

You’re playing with people’s lives, and don’t involve me in something and because even though you’re higher up and getting all this money, if it’s not right on that paper, I’m not gon’ be out here when you’re doing your pop-ups and your little news write-ups. …I will be one of the ones that call you on it.

Other members of the group reacted to these comments with nods and verbal agreement.

#### Theme 2: feeling used yet unseen: exclusion from decision-making processes

The LHAs were frustrated by the project’s premature ending; they were supposed to be involved for three years, and felt they were left out of the decision-making process. One LHA said, “We didn’t even get a chance to get it off the ground or make mistakes.” Another said, “It’s just so shocking” and they felt they had valuable suggestions on how the study could have been salvaged. The LHAs said they did not understand why the project wasn’t treated more like a pilot to “work out the kinks” and problem-solve, with the LHAs actively contributing.

The LHAs felt they had stepped up to help their community but did not feel heard or respected as community experts. Feeling frustrated, uninformed, and disconnected from the project’s leadership, they said, “They have a meeting every Friday. They sit around the roundtable, but it’s not… with us… the people who are actually out here doing [the work].” The LHAs felt the grant dollars drove decisions, and talked about how little power and influence they had since they were neither doctors nor those who had obtained the grant funding (“We don’t have the white coats”). They wished they had been included in conversations about the project’s strategies and future. The LHAs said they had high hopes at first that their knowledge would be helpful for the community, but that knowledge was not utilized. They said they would have liked to have crafted recruitment strategies based on their community expertise and training but could not do so. “We should’ve been able to recruit… *[Several agreed.]* We should’ve been able to get the patients ourselves**…**we should’ve been in charge of recruitment …getting out there to get them, instead of working off of a list.”

LHAs discussed patient recruitment enthusiastically; the LHAs felt most productive and useful during this stage. They wanted to hold events, create curricula, and develop topics for “pop-up sessions” (see Table 5). The LHAs felt prepared to recruit potential patients from their communities but were hamstrung by project requirements to use the RCT’s recruitment strategy which they asserted was ineffective. One said, “I’m in the community almost every single day and I do events almost every week, and that could’ve been a perfect setting to get people set up.” They said they would have posted flyers and recruited from the community directly rather than relying on hospital electronic medical records. One LHA explained, “Each one of us have expertise, skills and training and education in dealing with community,” and emphasized they had an impact in a short time. “We made a difference in two weeks” elaborating that they had already helped community members change eating habits and encouraged medication adherence.

**Table 5:** Session topics LHAs developed to work with patients

Topic name	Description/focus
Staying Active, Keep it Moving	Healthy eating and active living
Emotional Wellness, No Straight Jackets	Mental health and self-care
Yummy for My Tummy, Healthy and Delicious Cooking	Recipes and tips
Metro Health Alphabet Soup	Different medical disciplines and terms that relate to the care of hypertension patients
PTSD: Post Traumatic Slave Disorder	Recognizing the impact of history on African Americans
What’s in Your Hood?	Resources within the community
Family Matters	Family and social supports
Puff, Puff, Pass	Nicotine and marijuana’s influence on hypertension
Can I get a refill?	All about medications
Aging and Healthy Habits	Staying healthy as you age
Knowing the Signs: Your Body Matters	Recognizing symptoms
Environmental Barriers	Housing, furniture, food, employment, transportation, employment, and 211

#### Theme 3: worrying that project termination damaged their reputation

Ending the project after the LHAs had become invested in it and after they had established community members’ trust made them worry about damage to their standing in their community. The LHAs highlighted their commitment to the community. “I’m invested in the community. That comes first.” The LHAs wondered who would tell the community members with whom they had worked that they had moved on to another project and would no longer be championing the hypertension project. “We have connections with people in the community about this topic, and …some still call and say ‘What’s next? Are we still meeting?’” The LHAs said they had given community members information on reducing hypertension, and they still expected to learn about better controlling it. The LHAs strongly felt they could be helpful. “Some of the people, we made them aware, where they’ve been walking around with high blood pressure, that this is real serious.”

The LHAs built trusted community relationships, relationships that were important to them. They said, “We’re building a trust by giving them information and saying ‘Come on. Trust us for this program.’” They felt the project’s end would undermine those relationships and damage the trust they had built. The LHAs surmised the community members could be thinking (of them): “I finally had somebody who seemed like they care about me, and now it’s ended.” LHAs emphasized that they felt their positions in the community could be jeopardized and they would be held accountable. “Not only will we run into them, they’re gonna remember us. …They keep in touch with us.”

The LHAs also said they represented the project because research staff did not go into the neighborhoods where the LHAs worked and did not fully understand the fallout from the project’s ending. “I don’t think they really realize what impact that they’ll really do when you just drop it.” The LHAs were discouraged in part because they were connectors between the community and the researchers and “the brunt of it comes back on us.” One LHA described how they had reserved community space for the project.

I had one site and they set aside an office just for our project, and so the person that gave me the office, she called me a couple of weeks ago. She’s like “Are you guys still coming out here? Because we still got the office.”

The LHAs felt uninformed about the project, uncertain, and uncomfortable about running into people who wanted a project update. They wanted to notify the sites about the project’s transition and no longer needing the space which would have helped with closure.

#### Theme 4: disengaging from research

Burdensome institutional requirements, feeling used, and not feeling seen or appropriately valued were all very problematic and made the LHAs skeptical about contributing to the research process. As a result of their experiences, some LHAs asserted that community members need to consider disengaging from research altogether. “We have to stop coming to your table.…Stop coming to projects. When they ask you in the doctor’s office ‘Do you want to be a part of a study?’ ‘No. No. No. I don’t.’” One said:

Until we remove ourselves and start taking care of ourselves better and just say “Scrap these institutions.” …I mean obviously there are some people that are sick enough that need to come, but …we’re all capable of teaching people in our community.

Another participant summed up their feelings, saying the experience had been discouraging, and they resented having taken time away from their families to engage in the work only to end up feeling they had become part of systems oppressing African Americans. This was especially upsetting because the research purported to address African American health disparities. “Whatever the new cause is where the money is directed, we are put in a position where we think we’re being cared for and cared about, and at the end of the day, that’s just not what it is.” One LHA said if it was usual practice for projects to switch directions, they were skeptical about helping with research in the future.

### LHAs’ recommendations for effectively conducting community-engaged research

The LHAs made several recommendations that should be considered for future work utilizing LHAs in community-engaged research.

#### Provide a structured training curriculum

Although the LHAs acknowledged learning much about hypertension and medications, they said the training “wasn’t too structured or formal,” and that the curriculum “was formed as we went along” and they would have appreciated having a more structured curriculum. They also said they would have liked to have had more specific information about evidence-based practices. “I expected us to… use evidence-based medicine, or evidence-based information about hypertension,” noting that was a focus of their CHW training. The training, they said, was too short and should have been longer, and slower paced. One said: “As far as us getting together and gathering more information, it was kind of rolling, you know, kind of fast-paced.”

#### Develop trusting relationships

The LHAs had ideas for how to improve participants’ experiences. The LHAs emphasized the importance of building trust with participants and described developing trusting, strong relationships with research participants over time and with effort. LHAs also developed trust with the research participants by helping them get to and then navigate the large hospital’s footprint, from ensuring participants were parking in the appropriate place, to helping them find the data collection locations. “They would call us before they would call the hospital to ask where they need to go. That’s how connected we were after just that one phone call.”

It shows how effective and how great we were… when it came from us that’s out here in the community and dressed like them, not dressed in scrubs… they opened up, and they really listened, ‘cause they felt we cared and stuff. We made that difference.

The LHAs described efforts to convince participants to trust them, saying when they initially cold-called patients from their own phones, participants were skeptical and sometimes called the hospital to make sure the LHAs really were calling on the hospital’s behalf. Another participant said they had to work to convince patients why they should participate, but because they “believed wholeheartedly” in the study, this was not difficult.

#### Collect data in the community

The LHAs said that participants worried that if they participated in the study, they would be prescribed new medications they did not want. The LHAs said that having the data collected at the hospital inspired this fear. The LHAs said that participants’ experiences could be improved by collecting data within the community rather than requiring participants to come to the hospital. One said: “Just leave the whole process out in the community.” Discussing the various structural barriers such as transportation to the hospital, parking, and difficulty getting around the hospital, the LHAs said participants were often frustrated. “And then making sure they parked in the correct lot to get reimbursed, ‘cause you could get your parking stamped, taking public transportation, of course, that’s always a barrier.” If data collection in the community was not possible, the LHAs said that keeping clinical data collection staff consistent would help develop and maintain trust in the research.

## Discussion

The results discussed here reflected LHAs’ experiences related to the training they received as well as their overall experiences with assisting with recruitment in a research study that ended halfway through its expected tenure. The LHAs in this study felt they learned much from their training, felt confident in transmitting the information they learned, and wanted to be kept up to date on the latest information on hypertension [25]. They were passionate about hypertension, wanted to make a difference in their communities, and felt armed to do so with new knowledge from their training. However, their experiences with the study itself were of systemic and institutional barriers and feeling unseen and powerless. LHAs were frustrated by what they saw as unnecessary and burdensome demands research required, felt that despite their training and community connections, they had little input in how the research was carried out, and their expertise in the community was neither recognized nor utilized. By being involved with the research, they had hoped to apply their knowledge in the community through curriculum development, patient interaction, and relationship building. However, their experiences with the demands of research reflected the gaps between community and research, seeing elements of the research design (meant to ensure rigor) and research procedures as imposing unnecessary barriers and hampering recruitment.

Consistent with previous work, the LHAs saw the need to comply with the IRB, effort reporting, and other institutional requirements as burdensome, unnecessary, and not what they had signed up for. The LHAs, were, however, paid for the time they spent on these tasks. In some weeks, these were the only activities the LHAs could be paid for, because the actual study tasks were moving slowly. In this study, the requirement specifically to obtain a flu vaccine was a good example of one such burdensome requirement. The hospital in which the LHAs were based required all employees and contractors to receive the vaccine. African Americans have been found to have more vaccine hesitancy than other groups [34, 35], and in this study the LHAs did not see this as a reasonable requirement. However, as found in other studies, the LHAs also appreciated the resources available to them through the project’s university connections [25].

Overall, our results are consistent with earlier studies regarding the factors that facilitate and hinder the collaborative process and outcomes [36, 37]. Specifically, the LHAs did not fully trust the researchers and felt they were not appropriately respected and communicated with, might not have fully understood or accepted their roles, or understood the reasons for the project changes [36, 37]. Their training may have prepared them to be more active in their interactions with the patient research participants than their role demanded. However, the LHAs stimulated community involvement, reported being supportive and helpful to potential research participants, were active and enthusiastic collaborators, had built community relationships specific to the project, and developed recruitment strategies [36]. The results are also consistent with Shelton et al.’s contextual and organizational factors affecting program impact and sustainability [25]. Policies, partnerships, and external funding sources such as the NIH’s demands for research rigor affected how project leadership felt the project should be implemented and who was responsible for delivering the project’s intervention while the LHAs felt the project should have been more easily adaptable to community needs and strategies. The project might have benefited by better explaining the differences between clinical trials and community-based participatory research (CBPR) and how grant funding can constrain project decisions.

Our study also supports previous research that African Americans tend to mistrust academic researchers and medical interactions [38]. Scharff et al. argue that mistrust creates “a significant emotional burden” that, combined with persistent, everyday experiences of racism and discrimination within and outside medical spaces, contribute to a reluctance to trust [38]. It is possible our participants experienced distress, even peripheral trauma [14] from their experiences. The LHAs and participants in Scharff et al. also saw researchers’ and medical professionals’ primary motivations as financial [38]. This study attempted to mitigate mistrust among the Arrican American RCT study participants by ensuring both the academic research staff and LHAs were African American﻿. Despite attempts to increase trust, LHAs’ nevertheless mistrusted the research overall. Their mistrust was borne through their experiences with exclusion, institutional burdens, feeling used, and the concern that their reputations and job prospects, rather than being burgeoned by the experience, might be worsened. Our LHAs’ worries that their reputations may be damaged may be reasonable, given that community members can see them somewhat as outsiders, given their association with large institutions [16]. It is especially important to consider this work because minoritized groups’ disengagement from research could have dire consequences for improving health disparities, and researchers must do better to demonstrate they are worthy of trust.

### Implications and lessons learned

#### Improving community engaged research efforts

If we expect minoritized communities to participate in research, we must recognize how research and practice maintain and perpetuate inequities and be intentional about demonstrating our trustworthiness [38]. To ensure mechanisms are created to enhance the research team’s trustworthiness in the future, this study’s results were immediately shared with the project team (PI, coordinator, and research assistant) as well as the team at the clinical research unit where participant data were collected. The Center for Reducing Health Disparities team met to debrief and discuss where it fell short in this project, and how to be more trustworthy in the future. They also discussed how to better communicate RCTs’ restrictions and requirements, and why it is important to meet recruitment goals and comply with research funder requirements. The research team also offered explanations and clarification on issues the trainees brought up. For example, although the LHAs described challenges with “cold calling” participants, the team clarified that although the treatment group patient participants did not necessarily personally know the LHAs, they had been informed that they would be contacted by LHAs through both letters to their home and letters to their physicians. However, the LHAs were not involved in the process until the call which highlights why it is important to consider their perspectives. Second, although it is accurate that the project did not reimburse LHAs’ mileage expenses, it did pay them for travel time. However, future studies should consider reimbursing gas and other similar costs.

#### Ensure community members engaged in research get adequate training

Community members need frequent and adequate communication as well as a full understanding of research requirements when they are engaged in research efforts. Research has found community members can be effectively trained to better understand, engage in research, and be amenable to learning [39, 40]. Although most of the LHA training included topics on hypertension, much less focused on different types of study designs and distinctions between research approaches. When community members are involved in advanced study designs, such as RCTs, more focused and specific training might be necessary so that requirements like the IRB and particular recruitment processes are fully appreciated. Researchers considering using LHAs in research recruitment might consider what might need to be added to their primarily practice-based roles to smooth the transition to researchers.

Educating community members about research designs and evidence-based interventions could have helped the LHAs understand the need for consistency in delivering their interventions and the need to follow particular procedures. This, however, may point to a departure between community-based research and its focus on tailoring services and interventions, as compared to the demands imposed by the scientific method. The need for blood pressure, a key study outcome, to be measured with precision and consistency in a clinical setting did not seem to be fully understood, or not accepted as valid in this study. This issue might have been avoided if LHAs had been closely connected with a strong project leader and/or project “champion” as noted in past LHA research [25]. However, researchers must attend closely to the tensions between research goals and what is necessary for building and maintaining strong collaborative and equitable community relationships [41], as well as staying flexible and open to LHAs’ ideas [1]. This study suggests the need for continued vigilance, even among, or perhaps especially among, health disparities researchers in balancing research rigor with community concerns in community-engaged research.

#### Establish clear communication, expectations, and roles

It is crucial to ensure that community members fully understand the research process, including requirements, timelines, decision-making processes, and team member expectations. Future projects might lessen tensions by being extremely clear about expectations about the research process [42], specifically, time commitments and research roles and requirements, especially for RCTs (e.g., what must be done to ensure research rigor). It is also important to keep community members involved in research, updated on project changes, and allow them to contribute to strategizing about how to adapt to changes. Having such clarity and updates may have reduced the frustration and disappointment the LHAs experienced.

#### Respect community members’ perspectives and expertise

Although high-quality, rigorous research is important, it is equally important to fully include community members in the research process. We must value non-academic community expert knowledge and experience in working toward anti-racist practices in research focused on and with communities. Our LHAs wanted the project’s leadership to recognize their contributions, expertise, and ideas. Future projects should recognize the benefits of more fully integrating the community into research, including research design, particularly when they play a crucial role in the research and giving them agency, including decision-making. Having LHA representatives meet regularly with the research team might have helped avoid some tensions experienced in this project. Prior work in this area indicates LHAs might have been helpful as full research team members [3, 20] as they had ideas on how best to recruit participants given their strong connections in the community and eagerness to help. CBPR is an ideal framework for this, as it “seeks to identify and build on strengths, resources, and relationships that exist within communities” [43]. It is essential for academic researchers to take community members’ ideas seriously and ensure community members do not feel their ideas are dismissed or not valuable.

#### Limitations

One limitation is that the design was not CBPR. CBPR has been lauded as one way to encourage research/community collaboration and “establish trust, share power, foster co-learning, enhance strengths and resources, build capacity, and examine and address community-identified needs and health problems” [44, 45]. The study we have discussed here, while clearly engaging community members, both as participants and assisting with the research, was not CBPR. In this study, academic researchers designed the study and determined the methodological approach before the community members were brought aboard [8, 9]. It is possible this study would have been more successful if it had employed a true CBPR approach, making the LHAs equal partners. CBPR models that foster intentional, authentic partnerships increase trust, partnership synergy, and knowledge exchange [10, 36].

Another important limitation was that the LHAs were notified about the project’s discontinuation the day the focus group was held. The focus group was scheduled somewhat hurriedly after the facilitator was notified about the project’s end, to at least capture lessons learned from the training experiences. The facilitator reported that although LHAs were eager to share their perspectives and experiences, the overall tone of the focus group was negative. This might not have been the case if the focus group had been held immediately after the training, or even a more distant date from the announcement of the project's end. The focus group probably captured the LHAs’ most intense and immediate emotions about the project ending. They’d had little time to process the information, and these circumstances could have shaped their perceptions and feedback. Future research in this area should consider evaluating the training immediately after its end to capture trainees’ experiences and consider exploring how the training prepared participants for the research project later in the research process. The fact that the focus group was held when it was might mean important information about the training was forgotten, and the LHAs’ perspectives were influenced by the more current information about the project’s end.

It is also important to keep in mind that the data were collected in a focus group, so the LHAs might have not only fed off one another’s comments but also could have held back from being honest due to speaking in a group setting and concerns about confidentiality. However, that did not appear to be a concern; the participants appeared honest, and they talked about their experiences openly. Another limitation to consider is that the data were coded by one team member (the same person who facilitated the focus group), and this might have introduced bias in the data interpretation. However, we attempted to guard against bias by sharing the full data report with four project team members and one LHA, none of whom offered substantive feedback or disagreed with interpretations and/or conclusions. Only one LHA agreed to review the manuscript, and although they did not offer any negative feedback, it is possible others would have.

## Conclusion

LHAs have the potential to help reduce health disparities, given their strong community presence, both as effective community educators and as research partners. Training as an LHA yielded positive outcomes in increasing their employment potential and their knowledge of hypertension. The opportunity also increased their motivation to engage in future opportunities for them to make a difference in their community around hypertension. Participating in research, however, can be a mixed experience. Future programs should consider developing frequent communication mechanisms linking community stakeholders with research leadership to guard against the LHAs’ feeling exploited. Incorporating LHA feedback on how best to recruit participants would address research needs while maximizing community collaboration. Finally, even programs and research centers focusing on community-based solutions to health disparities need to stay cautious about how they might be perpetuating systemic racism and failing to promote equity in pursuit of research rigor, and endeavor to be worthy of communities’ trust.

## Data Availability

Data are available on request.
